# Robust discrimination between closely related species of salmon based on DNA fragments

**DOI:** 10.1007/s00216-024-05724-9

**Published:** 2025-01-18

**Authors:** Debra Ellisor, Mary Gregg, Angela Folz, Antonio Possolo

**Affiliations:** 1 Chemical Sciences Division, National Institute of Standards and Technology, Hollings Marine Laboratory, 331 Fort Johnson Road, Charleston, SC 29412 USA; 2 Statistical Engineering Division, National Institute of Standards and Technology, 325 Broadway, Boulder, CO 80305-3337 USA; 3 Department of Physics, University of Colorado, 390 UCB, Boulder, CO 80309-3337 USA; 4 Statistical Engineering Division, National Institute of Standards and Technology, 100 Bureau Drive, Gaithersburg, MD 20899-8980 USA

**Keywords:** Barcoding, DNA, MUSCLE, Alignment, Nominal, Entropy

## Abstract

Closely related species of *Salmonidae*, including Pacific and Atlantic salmon, can be distinguished from one another based on nucleotide sequences from the cytochrome *c* oxidase sub-unit 1 mitochondrial gene (COI), using ensembles of fragments aligned to genetic barcodes that serve as digital proxies for the relevant species. This is accomplished by exploiting both the nucleotide sequences and their quality scores recorded in a FASTQ file obtained via Next Generation (NextGen) Sequencing of mitochondrial DNA extracted from Coho salmon caught with hook and line in the Gulf of Alaska. The alignment is done using MUSCLE (Muscle 5.2) [1], applied to multiple versions of each fragment perturbed according to the nucleobase identification error probabilities underlying the quality scores. The Damerau-Levenshtein distance was used to determine the genetic barcode of the candidate species that is closest to each aligned, perturbed fragment. The “votes” that the sampled fragments cast for the different candidate species are then pooled and converted into identification probabilities, using weights determined by the entropy of the fragment-specific identification probability distributions. This novel approach to quantify the uncertainty associated with measurements made using NextGen Sequencing can be applied to discriminate closely related species, hence to value-assignment for reference materials supporting determinations of the authenticity of seafood, for example, NIST Reference Materials 8256 and 8257 (Coho salmon) [2].

**Supplementary Information:**

The online version contains supplementary material available at 10.1007/s00216-024-05724-9.

## Introduction

The National Institute of Standards and Technology (NIST) has been developing reference materials (RMs) since 1910. These materials possess well-characterized properties for use in verifying the accuracy of specific measurements, supporting the development of new measurement methods, establishing the metrological traceability of measurement results, and promoting innovation and industrial competitiveness. Customers, including members of the food industry, utilize NIST RMs for research advancement and product development. In recent years, NIST has been expanding the scope of the food RM program to include products for food safety and determination of food authenticity.

Food fraud has become an area of increasing concern, costing the global food industry an estimated US$40 billion per year. Fraud can include the partial or complete substitution of a product or ingredients in a product, false claims about the nature, provenance, or manner of producing a product, or mislabeling of a product, all of which can challenge the authenticity of foodstuffs [3]. Generally, intentional substitutions are economically motivated. Less expensive or less desirable products are substituted for those that are more expensive or rare, with high-priced commodities such as wine, olive oil, spices, and seafood, being typically targeted.

Seafood is one of the most highly traded international commodities and is priced at import by weight, species, and provenance, which includes source (for example, whether wild-caught or aquacultured). For some seafoods, product source can be determined solely by genetic means. For example, in the US market, most shrimp species that are wild-caught are not aquacultured and aquacultured species are not also wild-caught. Therefore, the shrimp’s genetic identity alone can elucidate product source. However, other seafood species are available in both wild-caught and aquacultured varieties, so additional means of authentication are required (e.g., nutritive, elemental, or isotopic profiling) [4–6].

Forensic DNA profiling is one common type of analysis used to determine the authenticity of seafood [7]. In general, the product is subsampled, a region of the proposed organism’s genome is targeted using specific primers, and this region is amplified by polymerase chain reaction (PCR). The region of the genome that is targeted and how the fragments are treated following amplification varies greatly depending on the method employed [8].

The resulting fragments can be (i) visualized using gel or capillary electrophoresis for comparative analysis of fragment length to determine species identity [9], (ii) employed in phylogenetic analysis to ascertain relatedness [10], or (iii) used for sequencing the entire amplicon and searching the results against a public or proprietary database containing reference data from verified species [11].

Quantitative polymerase chain reaction (qPCR) has also been used, particularly in determinations of genetically modified organisms (GMOs), to calculate the percentage of a total product that is authentic, which aids in identifying unauthorized and authorized GMOs [12]. Though the need has been recognized, none of these methods include proposed approaches to evaluate the uncertainty surrounding the species identification.

Morphological assessment and molecular genetic sequen-cing with phylogenetic reconstruction are commonly used to assign species to seafood products. Additional verification methods, such as the molecular genetic analyses described herein, typically generate binary results, which indicate whether the material is or is not the declared or expected species, or, when qPCR is used, whether the material is a mixture of multiple species expressed as proportions [13].

Relevant information that should be considered for these purposes includes base-pair matching fidelity, and success in alignment against genetic barcodes available in databases like the Reference Standard Sequence Library for Seafood Identification (RSSL) maintained by the US Food and Drug Administration (FDA), or the Barcode of Life Data System (BOLD). Noting the decline of classical taxonomic expertise, Hebert et al. [14] concluded that “the sole prospect for a sustainable identification capability lies in the construction of systems that employ DNA sequences as taxon ‘barcodes’,” and established that “the mitochondrial gene cytochrome *c* oxidase I (COI) can serve as the core of a global bioidentification system for animals.”

Sequencing-based methods typically qualify the reliability of the identity of a base pair in the form of a *Phred* score that can be translated into the probability that the base pair will have been identified incorrectly [15, 16]. These scores can be leveraged to evaluate the confidence in the ability of the chosen method to make identifications based on comparisons between aligned DNA fragments and reference sequences (genetic barcodes) of vouchered specimens of the species of interest.

We have developed a novel method to quantify the confidence surrounding the ability of a representative NextGen Sequencing method to verify the genetic identity of RM 8256, Wild-caught Coho Salmon [2], which is a fresh frozen fish homogenate prepared from multiple individuals collected off the coast of Alaska, USA. This material was developed to aid in determinations of the nutritional value, safety, and authenticity of seafood. The resulting confidence evaluations, for the nominal property whose values are the species the material was drawn from, support metrological traceability to reference sequences, preferably from vouchered exemplars of the species archived at the National Museum of Natural History (Smithsonian Institution), at the California Academy of Sciences, or at other, similarly reputable institutions.

## Methods

The probabilistic identification procedure described herein selects the most likely species for the material from among a set of ten candidate, likely species, represented by the reference sequences of suitable exemplars. The candidate species considered in this study were as follows: *Oncorhynchus gorbuscha* (Pink salmon), *Oncorhynchus keta* (Chum Salmon), *Oncorhynchus kisutch* (Coho salmon), *Oncorhynchus mykiss* (Steelhead trout), *Oncorhynchus nerka* (Sockeye salmon), *Oncorhynchus tshawytscha* (Chinook salmon), *Parahucho perryi* (Sakhalin taimen), *Salmo salar* (Atlantic salmon), *Salmo trutta* (Brown trout), and *Salvelinus alpinus* (Arctic char). These species afford a fairly complete representation of the members of the *Salmonidae* family that are the closest relatives of Coho salmon, according to the cladogram that Shedko et al. [17, Fig. 3] developed for this family, based on mitochondrial DNA (mtDNA) data.

The nucleotide sequences from the cytochrome *c* oxidase sub-unit 1 mitochondrial gene (COI) for six selected, vouchered specimens of Pacific fish (all in the genus *Oncorhynchus*), and for one vouchered specimen of Atlantic salmon (*Salmo salar*) listed in the RSSL, comprise between 649 and 656 nucleotides. Similar sequences for specimens of Brown trout (*Salmo trutta*) and Sakhalin taimen (*Parahucho perryi*) from the BOLD, and Arctic char (*Salvelinus alpinus*) from GenBank, comprise 631, 655, and 648 nucleotides, respectively. We will refer to such sequences as “reference sequences.”

Fragments of mtDNA from RM 8256 were obtained by PCR and were sequenced via NextGen Sequencing. The mtDNA was fragmented and purified at NSF AuthenTechnologies with an Ion Shear™ Reagents Kit (Life Technologies, Carlsbad, CA, USA) for a 400-base read library. A standard DNA extraction protocol was applied involving chloroform extraction followed by ethanol precipitation using a QIAcube system (Qiagen, Hilden, Germany) to ensure high purity and high yield. Samples were incubated in a heat block set to 37 $^{\circ }\text {C}$ for 8 min for a median fragment size of 350–450 base pairs. DNA ligase and nick repair polymerase were used on the fragmented sample DNA. To size select for a 400-base-read library, SizeSelect™ 2 % gel (Invitrogen, Waltham, MA, USA) was used in an iBase E-Gel unit (Invitrogen, Waltham, MA, USA) on top of a blue light transilluminator. The libraries were amplified and purified using Library Amplification Primer Mix and Platinum™ PCR SuperMix High Fidelity reagents (Invitrogen, Waltham, MA, USA). AmPure XP Reagent (Beckman Coulter, Brea, CA, USA) was added, which utilizes an optimized buffer selectively to bind DNA fragments 100 base pairs and larger to paramagnetic beads. Excess primers, nucleotides, salts, and enzymes were removed using a washing procedure that increased the purity of the PCR product. DNA concentration was determined using a Qubit Fluorometer unit, and the Ion Chef and Ion Torrent Personal Genome Machine were used for whole genome sequencing (ThermoFisher Scientific, Waltham, MA, USA), which yielded the raw FASTQ file used in this study.

The mtDNA fragments, of which 52,115 were sequenced for RM 8256, have lengths ranging from 25 to 495 nucleotides. A set of 100 fragments between 100 and 200 nucleotides long was drawn, uniformly at random, from the sub-population of 4839 fragments of that length, as described in the “Inputs” section. We will refer to these sequences as “sample sequences.” The “Results and discussion” section discusses motivations for this choice of sub-population.

A “perturbed” replicate of each fragment in this sample was generated that takes the site-specific identification uncertainty (attributable to potential errors in the identification of individual nucleotides) into account, and an ensemble of high-accuracy alignments to each of the aforementioned reference sequences was produced for each perturbed replicate, both as described in the “Uncertainty propagation throughalignment” section, and the Damerau-Levenshtein (DL) [18, 19] distances between the aligned version of the replicates and each of the reference sequences were computed. The DL distance between two sequences of characters (in the present setting drawn from the set $\{\text {A}, \text {C}, \text {G}, \text {T}\}$ denoting nucleotides) is the minimum number of insertions, deletions, or substitutions of a single character, or transpositions of two adjacent characters, that are needed to transform one sequence into the other [20, Example E6]. The two sequences being compared can be of different lengths, as they are in this application.

The resulting set of DL distances was used to determine how each fragment in the sample casts votes for the different species under consideration, and the results are pooled and summarized into a weighted average for each reference sequence separately, with weights that are decreasing functions of the entropy of the discrete probability distributions that describe the NextGen Sequencing uncertainty for each selected fragment, as described in the “Identification” section.

This procedure, detailed below, was implemented in the R language [21], which invokes Muscle 5.2 [1]. The R code is provided as Supplementary Material. It uses facilities implemented in R package stringdist [22].

### Inputs

The inputs to the proposed procedure were as follows:

- A FASTQ file [23] with the measured nucleotide sequen-ces of 52,115 fragments of mitochondrial DNA, and with the corresponding, site-specific quality scores, for NIST RM 8256, amounting to almost 15 million measured individual nucleotides.
- The reference sequences of *O. gorbuscha* (Pink), *O. keta* (Chum), *O. kisutch* (Coho), *O. mykiss* (Steelhead), *O. nerka* (Sockeye), *O. tshawytscha* (Chinook), and *S. salar* (Atlantic), from vouchered specimens listed in the US FDA’s RSSL database, plus reference sequences for specimens of *S. trutta* (Brown trout) and *P. perryi* (Sakhalin taimen) listed in the BOLD database, and *S. alpinus* (Arctic char) listed in the GenBank database.
- Specification of the number of fragments to use for the identification (100 in this study), range of their numbers of nucleotides (100–200), number of replicates of each (25), and size of the ensembles (16) of high-accuracy alignments to each reference sequence produced by Muscle 5.2 for each replicate of each selected fragment.

**Fig. 1:**
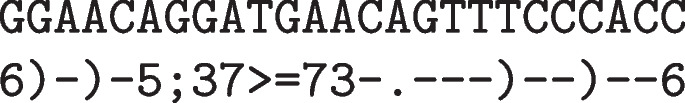
Sequence of measured nucleotides in a fragment of mtDNA of NIST RM 8256 (A $=$ adenine, C $=$ cytosine, G $=$ guanine, T $=$ thymine), and ASCII codes representing the corresponding *Phred* quality scores

### Uncertainty propagation through alignment

Figure 1 shows the sequence and the corresponding *Phred* quality score codes for a fragment (of length 25, chosen just for expository convenience) from RM 8256. These quality scores [15, 16] are specified as integers represented by ASCII (American Standard Code for Information Interchange) characters in the corresponding FASTQ file. The character representing a *Phred* score of *S* has ASCII code $S+33$. For example, the second quality score code for the string listed in Fig. 1, “),” has ASCII code 41, hence represents a Phred score of $41-33=8$, which translates into an error probability of $10^{-8/10}=0.16$. The article of the Wikipedia titled “ASCII” includes a table with all the ASCII codes.

The two sources of measurement uncertainty whose contributions were quantified and propagated to the final uncertainty were as follows: (i) the uncertainty that derives from errors in the identification of individual nucleotides and (ii) the uncertainty associated with the alignment of mtDNA fragments against each of the ten reference sequences under consideration.

**Fig. 2:**
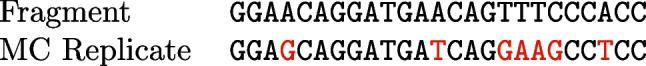
The first line has the same sequence listed in Fig. 1 as it was measured, and the second line has one of the 25 replicates that were generated for it based on the site-specific identification error probabilities. This particular replicate differs from the measured sequence in the seven sites marked in red

**Fig. 3:**
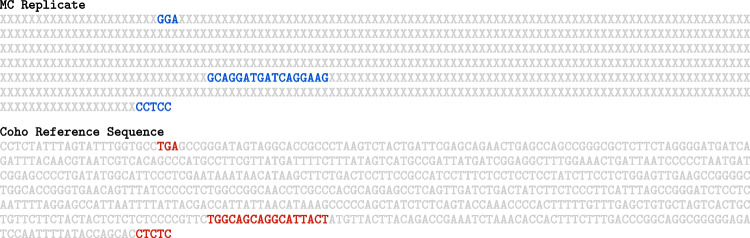
The top section labeled “MC Replicate” has the “perturbed” sequence listed in the second line of Fig. 2, from one of the 16 versions of the ensemble of the high-accuracy alignments that Muscle 5.2 produces in this case. The bottom section labeled “Coho Reference Sequence” has the 654-nucleotides long reference sequence for Coho salmon. The “X” indicate “missing” sites in the replicate. The DL distance between these two sequences, disregarding all these “missing” sites, is 10

These sources of uncertainty were evaluated using the random sample of 100 sequences that was drawn from the FASTQ file for RM 8256, as explained in the “Methods” section. Evaluating the joint contribution from both (i) and (ii) involved the following steps:

1. Twenty-five replicates of each fragment were generated so that if the error probability at a particular site is *p*, then the letter that was measured there (one of A, C, G, and T) is retained with probability $1-p$, and with probability *p*, it is replaced with one of the other three letters (each of which had probability *p*/3 of being selected as the replacement). Figure 2 shows the same fragment depicted in Fig. 1, and one of its replicates generated as just described.
2. For each such replicate, an ensemble comprising 16 versions of the high-accuracy alignment to each reference sequence were generated using the “diversified” option of Muscle 5.2, which maximizes the diversity of the biases of the alternative alignments, especially systematic errors [1, Table S1], thus helping mitigate such biases in downstream data reductions. Figure 3 shows one of the 16 versions of the high-accuracy alignment of the “perturbed” sequence depicted in the second line of Fig. 2, against the reference sequence for Coho salmon.

The end result of these steps is a collection of $100 \times 25 \times 16$ = 40,000 alignments against each reference sequence. Refer to the“Results and discussion” section for a discussion of the choices of numbers of replicates (25) and of the size (16) of the ensembles of high-accuracy alignments that were made.

### Identification

Table 1 lists the DL distances between each alignment in an ensemble of 16 high-accuracy alignments of the “perturbed” sequence listed in the second line of Fig. 2, and the reference sequences of the ten species being considered as potential sources for the mtDNA fragment listed in Fig. 1.

**Table 1:** DL distances between each of the alignments $a_{1}, \dots , a_{16}$ in the ensemble of 16 high-accuracy alignments of the “perturbed” sequence listed in the second line of Fig. 2, and the reference sequences of each of the ten species being considered as potential sources for the mtDNA fragment presented in Fig. 1

	$a_{1}$	$a_{2}$	$a_{3}$	$a_{4}$	$a_{5}$	$a_{6}$	$a_{7}$	$a_{8}$	$a_{9}$	$a_{10}$	$a_{11}$	$a_{12}$	$a_{13}$	$a_{14}$	$a_{15}$	$a_{16}$
*Oncorhynchus gorbuscha*	10	12	12	12	10	10	11	11	12	12	9	11	10	12	11	10
*Oncorhynchus keta*	8	11	10	11	10	10	6	10	11	9	7	7	10	9	10	8
*Oncorhynchus kisutch*	6	7	7	9	10	9	4	11	9	6	6	6	6	7	9	5
*Oncorhynchus mykiss*	9	12	8	11	9	9	9	8	10	11	9	9	10	9	8	10
*Oncorhynchus nerka*	7	13	8	12	8	9	10	9	11	12	9	9	10	8	9	10
*Oncorhynchus tshawytscha*	8	13	9	11	9	11	10	10	10	11	9	9	10	9	9	10
*Parahucho perryi*	10	12	10	10	9	10	11	10	10	10	9	9	11	10	11	10
*Salmo salar*	11	12	11	11	10	11	10	12	10	11	10	10	12	10	11	10
*Salmo trutta*	10	10	9	10	9	10	9	9	9	10	9	10	9	10	9	9
*Salvelinus alpinus*	9	13	9	15	9	9	10	10	10	12	9	10	13	12	11	11

For example, the DL distance between alignment $a_{1}$ and *O. gorbuscha*’s reference sequence is 10. Note that for some of the alignments listed in Table 1, there are several reference sequences for which the aligned sequence achieves the same shortest DL distance. For example, the shortest DL from $a_{6}$ to the ten reference sequences is 9, and it is achieved for *O. kisutch*, *O. mykiss*, *O. nerka*, and *S. alpinus*. Such ties are broken at random by slightly jittering these distances, using the default settings of function jitter that is implemented in the R environment for statistical programming and graphics [21].

Let *D* denote the table shown in Table 1, with 10 rows and 16 columns. For each column *j* (where $j=1,\dots , 16$), we find the reference sequence *i* (where *i* denotes a row number, $1 \leqslant i \leqslant 10$) for which *D*(*i*, *j*) (the distance between $a_{j}$ and reference sequence *i*) is the smallest of all the 10 entries in column *j*, with ties within each column broken as just explained.

The result is a sequence of 16 reference sequences, one per column: $(\text {O. kisutch }$, $\text {O. kisutch }$, $\text {O. kisutch }$, $\text {O. kisutch }$, $\text {O. nerka }$, $\text {O. kisutch }$, $\text {O. kisutch }$, $\text {O. mykiss }$, $\text {S. trutta }$, $\text {O. kisutch }$, $\text {O. kisutch }$, $\text {O. kisutch }$, $\text {O. kisutch }$, $\text {O. kisutch }$, $\text {O. mykiss }$, $\text {O. }$ $\text {kisutch })$. This means that *O. kisutch* is the closest match for 12 of the alignments, *O. mykiss* is the closest match for 2 alignments, and *O. nerka* and *S. trutta* are the closest matches for 1 alignment each.

Now, we repeat this process for the 25 replicates of the same fragment that express the site-specific uncertainty in the measurement of the nucleotides, meaning that we obtain 25 tables like the *D* in Table 1, from which we obtain $25 \times 16 = 400$ votes that this fragment casts for the ten species under consideration. Since we use 100 fragments, selected at random from among those in the FASTQ file for RM 8256 that comprise from 100 to 200 nucleotides, once we are done going through all of them, we have 100 rows each of which has a particular distribution of 400 “votes.” Table 2 shows the first five and the last five rows of the 100 rows.

**Table 2:** The first five and the last five rows of a table of votes with 100 rows corresponding to the selected fragments (F)

F	Og	Oke	Oki	Om	On	Ot	Pp	Ss	St	Sa	entropy /nat
1	0	0	400	0	0	0	0	0	0	0	0.000
2	0	0	248	2	0	150	0	0	0	0	0.691
3	0	0	400	0	0	0	0	0	0	0	0.000
4	0	0	400	0	0	0	0	0	0	0	0.000
5	0	0	400	0	0	0	0	0	0	0	0.000
$\vdots$	$\vdots$	$\vdots$	$\vdots$	$\vdots$	$\vdots$	$\vdots$	$\vdots$	$\vdots$	$\vdots$	$\vdots$	$\vdots$
96	0	0	400	0	0	0	0	0	0	0	0.000
97	0	0	400	0	0	0	0	0	0	0	0.000
98	0	0	400	0	0	0	0	0	0	0	0.000
99	0	0	399	0	0	1	0	0	0	0	0.017
100	0	0	400	0	0	0	0	0	0	0	0.000

Each row lists the number of votes that each fragment casts for each of the ten reference sequences, as being the best match for it. The total number of votes in each row is 400, which is the product of the number of replicates (25) generated for each fragment, and the number of high-accuracy alignments (16) in the ensemble that Muscle 5.2 generates for each replicate. In the header, the names of the ten species being considered as potential sources for the selected fragments of the mtDNA of RM 8256 were replaced by the following abbreviations: *Og*, *O. gorbuscha*; *Oke*, *O. keta*; *Oki*, *O. kisutch*; *Om*, *O. mykiss*; *On*, *O. nerka*; *Ot*, *O. tshawytscha*; *Pp*, *P. perryi*; *Ss*, *S. salar*; *St*, *S. trutta*; and *Sa*, *S. alpinus*. The last column lists the values of the entropy of the ten probabilities that are obtained when the entries in each row are each divided by 400

**Table 3:** Probabilities of the alternative species under consideration for NIST Reference Material 8256, based on 100 randomly selected fragments comprising between 100 and 200 nucleotides each

species		prob (%)	lwr (%)	upr (%)
*Oncorhynchus gorbuscha*	(Pink salmon)	0	0	0
*Oncorhynchus keta*	(Chum salmon)	0	0	0
*Oncorhynchus kisutch*	(Coho salmon)	97.7	94.7	99.7
*Oncorhynchus mykiss*	(Steelhead salmon)	0.151	0.0396	0.308
*Oncorhynchus nerka*	(Sockeye salmon)	0.00237	0	0.00721
*Oncorhynchus tshawytscha*	(Chinook salmon)	0.267	0.0738	0.543
*Parahucho perryi*	(Sakhalin taimen)	0.00473	0	0.0144
*Salmo salar*	(Atlantic salmon)	0	0	0
*Salmo trutta*	(Brown trout)	1.89	0	4.75
*Salvelinus alpinus*	(Arctic char)	0	0	0

The columns labeled “lwr (%)” and “upr (%)” list the endpoints of 95% confidence intervals for the probability estimates listed under “prob (%),” obtained by application of the non-parametric statistical bootstrap

Most of the selected 100 fragments cast all their 400 votes for Coho, hence assigning probability 1 to Coho and 0 to each of the other nine species. The corresponding entropy is $-(1 \times \log (1) + 9 \times 0 \times \log (0)) = {0 }{nat}$, the smallest that it can be. The nat is the natural unit of information, based on natural (base *e*) logarithms, which we use throughout.

Fragment 2, however, distributes its votes a bit more widely, even if it still favors Coho. The corresponding probability distribution places $248/400 = 0.62$ probability on Coho, $2/400 = 0.005$ on Steelhead, $150/400=0.375$ on Chinook, and 0 on all the others. The entropy of this probability distribution is $-((248/400)\times \log (248/400) + (2/400)\times \log (2/400)) + (150/400)\times \log (150/400)) = {0.691\,\textrm{nat}}$.

In summary, the more evenly distributed the unit of probability is over the ten species under consideration, the larger the entropy, hence the greater the ambiguity, or the smaller the discrimination acumen, of the corresponding fragment. It stands to reason that the votes the different fragments cast for each reference sequence (hence for the corresponding candidate species) should be weighed according to how informative they are about between-species differences. Since the entropy quantifies their informativeness, the weights should be a decreasing function of entropy.

The function we chose was determined based on an analogy with the relation between the entropy, *S*, and the standard deviation, $\sigma$, of a Gaussian distribution: $S = \frac{1}{2} \log (2\pi e) + \log \sigma$. When combining observations of the same quantity affected by errors with possibly different standard deviations, and the errors of observation are Gaussian with mean zero, a weighted average is often used, with weights proportional to the reciprocals of the squares of these standard deviations. In this conformity, we will assign a weight proportional to $\exp (-2S)$ to a fragment that produces an identification probability distribution with entropy *S*.

Let *V* denote the matrix with 100 rows and 10 columns whose entries are the vote counts listed in Table 2 (that is, the values in all its columns other than the first and the last), and let *w* denote the vector of non-negative weights adding to 1, $w_{i} = \exp (-2S_{i})/(\exp (-2S_{1})+\dots +\exp (-2S_{100}))$ for $i=1,\dots ,100$, assigned to the selected fragments.

The vote counts in column *j* of matrix *V* are pooled into a weighted average and converted into the probability of each species being the source of RM 8256 as follows: $p_{j} = \sum _{i=1}^{100} w_{i}V_{i,j} / T$ with $j=1,\dots ,10$ denoting the species as listed in the header of Table 2, where $T = \sum _{j=1}^{10} \sum _{i=1}^{100} w_{i} V_{i,j}$. This calculation gives greater weight to the more informative fragments (those whose votes are predominantly concentrated on one species) than to the less informative ones (those others that distribute their votes more widely over the ten candidate species under consideration). The resulting $p_{1}, \dots , p_{10}$ are listed in Table 3.

## Results and discussion

Using these sample sequences, we were able to verify the species in RM 8256 as *Oncorhynchus kisutch* with 97.7% confidence (or sensitivity, which is the probability of correctly identifying the material as Coho salmon when in fact it is so). The true value of this sensitivity is believed to lie between 94.7 and 99.7%, with 95% probability, based on the non-parametric statistical bootstrap [24].

It should be noted that the procedure described herein does not purport to achieve an absolute identification (among all living species) of the source of RM 8256, using only the data in the FASTQ file containing the output of NextGen Sequencing of DNA extracted from the material. Implicitly, we rely also on highly informative, reliable substantive prior knowledge (of the fishermen and of the zoologists who will have inspected the fish) that the catch from the Gulf of Alaska that became RM 8256 indeed comprises only Coho salmon. Genetic analysis was done to corroborate morphological species identification, and this study does offer compelling verification that the chances are very small of the catch being from some other, closely related species, supporting the aforementioned prior knowledge overwhelmingly [25].

Table 3 lists the probabilities of the different species under consideration. The Shannon entropy [26] of this identification probability distribution is 0.12 nat: in this case, with ten possible species being considered alternatives for RM 8256, the entropy can take any value between the minimum of 0 nat (for the most concentrated probability distribution, which places probability 1 on one species and 0 on all the others) and the maximum of $-10\times (1/10)\times \log (1/10) = {2.3\,\textrm{nat}}$ (for the most dispersed probability distribution, which places the same probability on each of the candidate species).

While we did not explore exhaustively or systematically the effect of considering multiple reference sequences from different exemplars of the same species simultaneously, we did obtain results very close to those listed in Table 3 in those instances when multiple reference sequences for Coho salmon, or for several of the other species, were in the panel of alternatives under consideration. After aggregating the individual results by species, the probabilities of the different species were quite similar to those listed above, up to measurement uncertainty. This suggests that our approach to identity verification is robust to within-species genomic variability.

It is remarkable that such sharp discrimination between closely related species can be achieved with those few fragments whose average length is less than one-quarter of the length of the mtDNA region in COI that is typically used for fish identification. The aforementioned confidence can be increased further, at greater computational expense, by using a larger set of fragments, and also by selecting a wider range of lengths for the fragments than the range (100–200) used to reach 97.7% confidence.

This confidence level reflects both (i) the site-specific uncertainty that is reported in the FASTQ file listing the sequencing results for the fragments from RM 8256, as expressed in the corresponding quality scores, and (ii) the alignment uncertainty, evaluated using ensembles of high-accuracy alignments produced by Muscle 5.2 [1].

The following choices all are influential upon the results reported in Table 3:

1. The number of fragments to use (100)
2. The range (100–200) of the number of nucleotides that define the population of fragments to be sampled
3. The number of replicates (25) of each fragment that express the identification uncertainty of the nucleotide at each site of each fragment (based on the quality scores reported in the FASTQ file that lists all the fragments that have been sequenced)
4. The number (16) of alternative alignments in the ensemble of high-accuracy alignments that Muscle 5.2 generates for each replicate of each fragment
5. The manner of deciding what it means for a fragment to cast a vote for a particular species
6. The weight to assign to the set of votes cast by each fragment
7. The manner of pooling the weighted votes (we opted for the species-specific weighted averages: alternatives could have been the species-specific weighted median, or linear pooling [27, §6.3] of the identification probabilities of the different, selected fragments)

The DL distance between (each aligned version of each replicate of) each fragment and a reference sequence does not reflect either (i) the length of the fragment or (ii) the number and lengths of the gaps in the alignment (apparent in the example of Fig. 3). However, (i) is not very influential in our application because we restrict attention to fragments whose lengths are between 100 and 200 nucleotides, and (ii), which can potentially be a shortcoming of DL, is mitigated by this other fact: for the fragment depicted in Fig. 3, and for many other fragments, there are several very short, matched subsequences separated by considerable gaps, and one or two longer subsequences that are also matched. We conjecture that it is these longer, matched subsequences of the fragment that carry most of the information about the identification. If this conjecture is true, then this would imply that neither the number or extent of any gaps nor the composition of the aligned, very short subsequences, are particularly influential.

Increasing either the number of fragments to use (C1) or the average length of the fragments to sample from (C2), or both, generally will sharpen the discriminatory acumen of the procedure outlined above. However, this comes at a price of greater computational expense. The larger the average length of the fragments to sample from, the smaller and less varied the sub-population of fragments that is sampled. In this case, fragments 100–200 nucleotides long proved far more efficacious at discriminating Coho salmon from its close relatives, than fragments with lengths between 75 and 150 nucleotides. Still, 100 to 200 nucleotides amount to only 15 to 30% of the typical length of the reference sequences used in the process, revealing the acumen of this statistical approach to species discrimination and identification.

Both the number of replicates (C3) and the size of the ensemble of high-accuracy alignments generated for each replicate (C4) could have values larger than those that were chosen. On the one hand, choices of larger values for these two counts may expose uncertainties more thoroughly than the chosen values allow and likely will increase their contributions to the overall uncertainty. On the other hand, if the number of fragments sampled increases, too, the overall uncertainty will decrease. The choices made seem to strike a felicitous balance between accuracy and practicability.

**Fig. 4:**
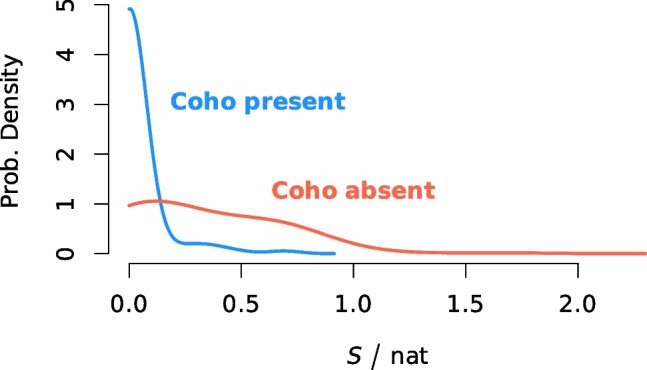
Probability density estimates for the entropy, *S*, of the 100 fragment-specific identification probability distributions in the case where Coho is among the candidate species under consideration (blue curve), and in the case where it is left out (red curve)

The question can be asked of what the procedure described in the “Methods” section does if the reference sequence for Coho salmon is left out, even if doing so would run counter to the trustworthy substantive knowledge in hand about the catch of fish whose tissue became this RM 8256. In such case, the procedure will still propose one of the other species as the most likely one for each fragment, because that is what it is designed to do. However, when Coho is left out, the distribution of the values of the entropy of the probabilities that the fragments assign to the different species changes dramatically, versus when Coho is included, as Fig. 4 shows. This serves as a clear warning that the correct species is not in the panel of candidate species under consideration.

When Coho is entertained as one of the candidate species, the probability density of the entropy values is sharply peaked at 0 nat, as the vast majority of the fragments do point to the correct species and only to the correct species. When Coho is left out, the procedure tries to make do with what previously were distant second or third best alternatives to Coho, and the resulting distribution of the values of the entropy flattens out markedly, suggesting that the fragments by and large are rather non-committal about which may be the best choice for the simple reason that what would have been the best choice is unavailable.

The approach we have described, based on NextGen Sequencing, is generic in the sense that it does not rely on PCR involving primers that target regions of the genome that are specific to particular genera or species. Neither does it involve high-resolution melting analysis as a follow-up to PCR. Fernandes et al. [28] review these and other alternative techniques that can be used to exploit identification information in reference sequences. Since our approach is generic, and involves only the data stored in a standard FASTQ file, it can readily be applied to a wide range of genome-driven identification tasks.

## Conclusion

The probabilities of the different species under consideration that are listed in Table 3 overwhelmingly verify RM 8256 as tissue of *Oncorhynchus kisutch*, which is consistent with the substantive prior knowledge about the catch, thus reinforcing and lending further credence to the prior belief that the source of that tissue was Coho salmon.

That such decisive conclusion can be reached using only 100 fragments whose average length is less than one-quarter of the length of the genetic reference sequence typically used to identify fish species is both surprising and remarkable, especially considering that the alternative species under consideration are closely related to Coho salmon and to one another taxonomically and genetically. This attests to the power of the statistical approach to verify species assignments based on NextGen Sequencing.

The confidence in such verification, 97.7% (but that most likely can be no lower than 94.7% and as high as 99.7%), expresses the following: (i) the natural variability of the genetic make-up of individual fish, to the extent that the material was derived from blended tissue of a catch of exemplars of wild-caught Coho salmon, not from a single individual, and given also that the population of fragments of length between 100 and 200 was sampled uniformly at random from the sub-population of fragments of such lengths; (ii) the uncertainty surrounding the measurement of the nucleotides at the different sites of each fragment (expressed in the corresponding quality scores and underlying error probabilities); and (iii) the alignment uncertainty that is inherent to the Muscle 5.2 alignment procedure.

## Supplementary Information

Below is the link to the electronic supplementary material.

Supplementary file 1 (pdf 122 KB)

Supplementary file 2 (pdf 149 KB)

## References

[1] Edgar RC. Muscle5: high-accuracy alignment ensembles enable unbiased assessments of sequence homology and phylogeny. Nat Commun. 2022;13:6968. 36379955 10.1038/s41467-022-34630-w PMC9664440

[2] Ellisor DL, Place B, Phillips M, Yen J. Analysis of seafood reference materials: RM 8256, RM 8257, RM 8258, and RM 8259, Wild-Caught Coho Salmon (RM 8256), Aquacultured Coho Salmon (RM 8257), Wild-Caught Shrimp (RM 8258), Aquacultured Shrimp (RM 8259) NIST Special Publication 260–214. Gaithersburg, MD: National Institute of Standards and Technology; 2021.

[3] Manning L, Soon JM. Food safety, food fraud, and food defense: a fast evolving literature. J Food Sci. 2016;81:R823–34. 26934423 10.1111/1750-3841.13256

[4] González-Domínguez R. Food authentication: techniques, trends and emerging approaches. Foods. 2020;9:346. 32191996 10.3390/foods9030346 PMC7142980

[5] González-Domínguez R. Food authentication: techniques, trends and emerging approaches (second issue). Foods. 2022;11:1926. 35804739 10.3390/foods11131926 PMC9265475

[6] Christopher SJ, Ellisor DL, Davis WC. Investigating the feasibility of ICP-MS/MS for differentiating NIST salmon reference materials through determination of Sr and S isotope ratios. Talanta. 2021;231: 122363. 33965029 10.1016/j.talanta.2021.122363

[7] Primrose S, Woolfe M, Rollinson S. Food forensics: methods for determining the authenticity of foodstuffs. Trends Food Sci Technol. 2010;21:582–90.

[8] Rasmussen RS, Morrissey MT. DNA-based methods for the identification of commercial fish and seafood species. Compr Rev Food Sci Food Saf. 2008;7:280–95. 33467804 10.1111/j.1541-4337.2008.00046.x

[9] Dooley JJ, Sage HD, Brown HM, Garrett SD. Improved fish species identification by use of lab-on-a-chip technology. Food Control. 2005;16:601–7.

[10] Bartlett SE, Davidson WS. FINS (Forensically Informative Nucleotide Sequencing): a procedure for identifying the animal origin of biological specimens. BioTechniques. 1992;12:408–11. 1571152

[11] Landi M, et al. DNA barcoding for species assignment: the case of Mediterranean marine fishes. PLOS One. 2014;9:1–9. 10.1371/journal.pone.0106135 PMC4164363 25222272

[12] Burns M. A perspective on quantitative DNA approaches. In: Burns M, Foster L, Walker M, editors. DNA Techniques to Verify Food Authenticity: Applications in Food Fraud Ch.9 (The Royal Society of Chemistry, London, UK); 2019.

[13] Burns M, et al. Measurement issues associated with quantitative molecular biology analysis of complex food matrices for the detection of food fraud. Analyst. 2016;141:45–61. 26631264 10.1039/c5an01392e

[14] Hebert PDN, Cywinska A, Ball SL, deWaard JR. Biological identifications through DNA barcodes. Proc R Soc Lond Ser B Biol Sci. 2003;270:313–21. 10.1098/rspb.2002.2218 PMC1691236 12614582

[15] Ewing B, Hillier L, Wendl MC, Green P. Base-calling of automated sequencer traces using Phred. I. Accuracy assessment. Genome Res. 1998;8:175–85. 9521921 10.1101/gr.8.3.175

[16] Ewing B, Green P. Base-calling of automated sequencer traces using Phred. II. Error probabilities. Genome Res. 1998;8:186–94. 9521922

[17] Shedko SV, Miroshnichenko IL, Nemkova GA. Phylogeny of salmonids (Salmoniformes: Salmonidae) and its molecular dating: analysis of mtDNA data. Russ J Genet. 2013;49:623–37. 10.7868/s0016675813060118 24450195

[18] Damerau FJ. A technique for computer detection and correction of spelling errors. Commun ACM. 1964;7:171–6.

[19] Levenshtein VI. Binary codes capable of correcting deletions, insertions, and reversals. Soviet Physics Doklady. 1966;10:707–10. Doklady Akademii Nauk SSSR, V163 No4 845-848 1965.

[20] Possolo A. Simple guide for evaluating and expressing the uncertainty of NIST measurement results (National Institute of Standards and Technology, Gaithersburg, MD); 2015. NIST Technical Note 1900.

[21] R Core Team. R: a language and environment for statistical computing. R foundation for statistical computing, Vienna, Austria; 2024. https://www.R-project.org/.

[22] van der Loo MPJ. The stringdist package for approximate string matching. R J. 2014;6:111–22.

[23] Cock PJ, Fields CJ, Goto N, Heuer ML, Rice PM. The Sanger FASTQ file format for sequences with quality scores, and the Solexa/Illumina FASTQ variants. Nucl Acids Res. 2010;38:1767–71. 20015970 10.1093/nar/gkp1137 PMC2847217

[24] Efron B, Tibshirani RJ. An introduction to the bootstrap (Springer-ScienceBusiness Media. Dordrecht: The Netherlands; 1993.

[25] Ellisor DL, et al. Multi-omics characterization of NIST seafood reference materials and alternative matrix preparations. Anal Bioanal Chem. 2024;416:773–85. 37723254 10.1007/s00216-023-04928-9

[26] Rioul O. This is IT: a primer on Shannon’s entropy and information. In: Duplantier B, Rivasseau V, editors, Information Theory: Poincaré Seminar; 2018 .p. 49–86 (Springer, Cham, Switzerland, 2021).

[27] Koepke A, Lafarge T, Possolo A, Toman B. Consensus building for interlaboratory studies, key comparisons, and meta-analysis. Metrologia. 2017;54:S34–62.

[28] Fernandes TJR, Amaral JS, Mafra I. DNA barcode markers applied to seafood authentication: an updated review. Crit Rev Food Sci Nutr. 2021;61:3904–35. 32838560 10.1080/10408398.2020.1811200

