# Publisher Correction: Robust discrimination between closely related species of salmon based on DNA fragments

**DOI:** 10.1007/s00216-025-05800-8

**Published:** 2025-03-06

**Authors:** Debra Ellisor, Mary Gregg, Angela Folz, Antonio Possolo

**Affiliations:** 1 Chemical Sciences Division, National Institute of Standards and Technology, Hollings Marine Laboratory, 331 Fort Johnson Road, Charleston, SC 29412 USA; 2 Statistical Engineering Division, National Institute of Standards and Technology, 325 Broadway, Boulder, CO 80305-3337 USA; 3 Department of Physics, University of Colorado, 390 UCB, Boulder, CO 80309-3337 USA; 4 Statistical Engineering Division, National Institute of Standards and Technology, 100 Bureau Drive, Gaithersburg, MD 20899-8980 USA

**Publisher Correction: Analytical and Bioanalytical Chemistry**

10.1007/s00216-024-05724-9

The publisher regrets that the article contained the following mistakes:

1. In this article the affiliation details for Author Debra Ellisor were incorrectly given as 'Biospecimen Science Group, National Institute of Standards and Technology' but should have been ‘Chemical Sciences Division, National Institute of Standards and Technology'.
2. In section “Method” the first chapter was incorrect:

The candidate species considered in this study were as follows: *Oncorhynchus gorbuscha* (Pink salmon), *Oncorhynchus kea* (Chum Salmon), *Oncorhynchus kisutch* (Coho salmon), *Oncorhynchus mykiss* (Steelhead salmon), *Oncorhynchus nerka* (Sockeye salmon), *Oncorhynchus tshawytscha* (Chinook salmon), *Parahucho perryi* (Sakhalin taimen), *Salmo salar* (Atlantic salmon), *Salmo trutta* (Brown trout), and *Salvelinus alpinus* (Arctic char).

Correct:

The candidate species considered in this study were as follows: *Oncorhynchus gorbuscha* (Pink salmon), *Oncorhynchus kea* (Chum Salmon), *Oncorhynchus kisutch* (Coho salmon), *Oncorhynchus mykiss* (Steelhead trout), *Oncorhynchus nerka* (Sockeye salmon), *Oncorhynchus tshawytscha* (Chinook salmon), *Parahucho perryi* (Sakhalin taimen), *Salmo salar* (Atlantic salmon), *Salmo trutta* (Brown trout), and *Salvelinus alpinus* (Arctic char).

3. In section “Method” the second chapter was incorrect:

The nucleotide sequences from the cytochrome *c* oxidase sub-unit 1 mitochondrial gene (COI) for six selected, vouchered specimens of Pacific salmon (all in the genus *Oncorhynchus*), and for one vouchered specimen of Atlantic salmon (*Salmo salar*) listed in the RSSL, comprise between 649 and 656 nucleotides. Similar sequences for specimens of Brown trout (*Salmo trutta*) and Arctic char (*Salvelinus alpinus*) from the BOLD, and Sakhalin taimen (*Parahucho perryi*) from GenBank, comprise 631, 648, and 655 nucleotides, respectively. We will refer to such sequences as “reference sequences.”

Correct:

The nucleotide sequences from the cytochrome *c* oxidase sub-unit 1 mitochondrial gene (COI) for six selected, vouchered specimens of Pacific fish (all in the genus *Oncorhynchus*), and for one vouchered specimen of Atlantic salmon (*Salmo salar*) listed in the RSSL, comprise between 649 and 656 nucleotides. Similar sequences for specimens of Brown trout (*Salmo trutta*) and Sakhalin taimen (*Parahucho perryi*) from the BOLD, and Arctic char (*Salvelinus alpinus*) from GenBank, comprise 631, 655, and 648 nucleotides, respectively. We will refer to such sequences as “reference sequences.”

4. In section “Methods” subsection “Inputs” the second subpoint was incorrect:

- The reference sequences of *O. gorbuscha* (Pink), *O. keta* (Chum), *O. kisutch* (Coho), *O. mykiss* (Steelhead), *O. nerka* (Sockeye), *O. tshawytscha* (Chinook), and *S. salar* (Atlantic), from vouchered specimens listed in the US FDA’s RSSL database, plus reference sequences for specimens of *S. trutta* (Brown trout) and *S. alpinus* (Arctic char) listed in the BOLD database, and *P. perryi* (Sakhalin taimen) listed in the GenBank database.

Correct:

- The reference sequences of *O. gorbuscha* (Pink), *O. keta* (Chum), *O. kisutch* (Coho), *O. mykiss* (Steelhead), *O. nerka* (Sockeye), *O. tshawytscha* (Chinook), and *S. salar* (Atlantic), from vouchered specimens listed in the US FDA’s RSSL database, plus reference sequences for specimens of *S. trutta* (Brown trout) and *P. perryi* (Sakhalin taimen) listed in the BOLD database, and *S. alpinus* (Arctic char) listed in the GenBank database.

5. In section “Method” subsection “Identification” the following part was incorrect:

Most of the selected 100 fragments cast all their 400 votes 328 for Coho, hence assigning probability 1 to Coho and 0 to 329 each of the other nine species. The corresponding entropy is 330 -(1 × log(1) + 8 × 0 × log(0)) = 0nat, the smallest that it 331 can be. The nat is the natural unit of information, based on 332 natural (base e) logarithms, which we use throughout.

Correct:

Most of the selected 100 fragments cast all their 400 votes 328 for Coho, hence assigning probability 1 to Coho and 0 to 329 each of the other nine species. The corresponding entropy is 330 -(1 × log(1) + 9 × 0 × log(0)) = 0nat, the smallest that it 331 can be. The nat is the natural unit of information, based on 332 natural (base e) logarithms, which we use throughout.

The original article has been corrected.

