# Supplementary material for: Robust discrimination between closely related species of salmon based on DNA fragments
Source: Anal Bioanal Chem. 2025 Jan 18;417(12):2579–88. doi: 10.1007/s00216-024-05724-9 (PMC12003528; doi:10.1007/s00216-024-05724-9)
Supplement: Supplementary file 1 — (pdf 122 KB) [file 216_2024_5724_MOESM1_ESM.pdf]

## Supplementary material

The supplementary material comprises data and R codes that can be used to reproduce the results gathered in section 3. The data for RM 8256 consists of a FASTQ file with all the sequences that are 100-200 nucleotides long, which are a subset of the sequences determined via NextGen Sequencing of RM 8256 (https://doi.org/10.18434/mds2-3578). The reference sequences are listed in Table 4, with sufficient identifying information to enable their retrieval from the online repositories hyperlinked under RSSL, BOLD, and GenBank in the caption of the same table.

**Table 4** Locations of the ten reference sequences used in this study. The abbreviations in the headers mean the following: ACC = Accession, REP = Repository, SEQ ID = Sequence ID, SP ID = Specimen ID, and VCHR = Voucher. The repositories are RSSL, BOLD, and GenBank

| EXEMPLAR | REP | SP ID | VCHR |
|---|---|---|---|
| *Oncorhynchus gorbuscha* | RSSL | FDA 245 | USNM 404986 |
| *Oncorhynchus keta* | RSSL | FDA 243 | USNM 404990 |
| *Oncorhynchus kisutch* | RSSL | FDA 244 | USNM 404991 |
| *Oncorhynchus mykiss* | RSSL | RFE 386 | CAS-ICH 84878 |
| *Oncorhynchus nerka* | RSSL | FDA 242 | USNM 404989 |
| *Oncorhynchus tshawytscha* | RSSL | FDA 241 | USNM 405749 |
| *Salmo salar* | RSSL | FDA 024 | USNM 394151 |
| EXEMPLAR | REP | SP ID | SEQ ID |
| *Parahucho perryi* | BOLD | JX232184 | GBGCA3923-13.COI-5P |
| *Salmo trutta* | BOLD | JN007797 | ANGBF7897-12.COI-5P |
| EXEMPLAR | REP | ACC | VCHR |
| *Salvelinus alpinus* | GenBank | KJ128605 | NRM60042 |

Robert Edgar generously makes the source code for Muscle 5.2 publicly available on GitHub. The corresponding executable that was used in this study was built under Linux Mint 22 “Wilma” (based on Ubuntu 24.04) from this source code. The procedure described in section 2 was implemented in the R language [21], which invokes the executable for Muscle 5.2.

The part of the procedure that produced the probabilities listed in Table 3 took 86 minutes to complete, running on an AMD Ryzen 7 5800H CPU clocked at 3.2 GHz to 4.4 GHz, with 8 cores and 16 threads, 6 megabytes of L2 cache, and 16 megabytes of L3 cache, but without explicit parallelization and without any other significant loads, under Linux Mint 22, with 32 gigabytes of available RAM.
