# Supplementary material for: Robust discrimination between closely related species of salmon based on DNA fragments
Source: Anal Bioanal Chem. 2025 Jan 18;417(12):2579–88. doi: 10.1007/s00216-024-05724-9 (PMC12003528; doi:10.1007/s00216-024-05724-9)
Supplement: Supplementary file 2 — (pdf 149 KB) [file 216_2024_5724_MOESM2_ESM.pdf]

# Robust discrimination between closely related species of salmon based on DNA fragments

Debra Ellisor[1]*, Mary Gregg[2], Angela Folz[2,3], Antonio Possolo[4]

[1]*Biospecimen Science Group, National Institute of Standards and Technology, Hollings Marine Laboratory, 331 Fort Johnson Road, Charleston, SC 29412, United States.

[2]Statistical Engineering Division, National Institute of Standards and Technology, 325 Broadway, Boulder, CO 80305-3337, United States.

[3]Department of Physics, University of Colorado, 390 UCB, Boulder, CO 80309-3337, United States.

[4]Statistical Engineering Division, National Institute of Standards and Technology, 100 Bureau Drive, Gaithersburg, MD 20899-8980, United States.

*Corresponding author e-mail: debra.ellisor@nist.gov

Contributing authors: mary.gregg@nist.gov; angela.folz@nist.gov; antonio.possolo@nist.gov

```
#######################################################################

## FILE NAME:         barcodes2024Oct19.R
## AUTHORS:           Debra Ellisor, Mary Gregg,
##                    Angela Folz, Antonio Possolo

#######################################################################
## SECTION (R + R PACKAGES + MUSCLE) ==================================
#######################################################################

## R version 4.4.1 (2024-06-14) -- "Race for Your Life"

## stringdist 0.9.12

## Muscle 5.2: Robert Edgar -- https://www.drive5.com/muscle
## Source code from https://github.com/rcedgar/muscle/releases/tag/v5.2

#######################################################################
## SECTION (BARCODES SOURCES) ========================================
#######################################################################

## Each file containing a barcode (reference sequence) is a
## hand-assembled, plain ASCII text file that may have one or several
## initial lines with metadata followed by a line whose first
## character is ">". The lines after this one have the actual barcode,
## which will have been retrieved from the sources listed below. The
## only characters allowed in the lines with the barcode are from the
## set {A,C,G,T} as well as blank spaces and (invisible) newline
## characters, all of which will be deleted

## Reference Standard Sequence Library for Seafood Identification (RSSL)
## https://www.fda.gov/food/dna-based-seafood-identification/reference-
standard-sequence-library-seafood-identification-rssl

##       "OncorhynchusGorbuscha-FDA245.txt"    ## Pink Salmon
##       "OncorhynchusKeta-FDA243.txt"         ## Chum Salmon
##       "OncorhynchusKisutch-FDA244.txt"      ## Coho Salmon
##       "OncorhynchusMykiss-RFE386.txt"       ## Steelhead Salmon
##       "OncorhynchusNerka-FDA242.txt"        ## Sockeye Salmon
##       "OncorhynchusTshawytscha-FDA241.txt"  ## Chinook Salmon
##       "SalmoSalar-FDA24.txt"                ## Atlantic Salmon

## Barcode of Life Database (BOLD)
## https://www.boldsystems.org

##       "ParahuchoPerryi-JX232184.txt"        ## Sakhalin taimen
##       "SalmoTrutta-JN007797.txt"            ## Brown Trout

## National Library of Medicine (NIH) -- GenBank Nucleotide
## https://www.ncbi.nlm.nih.gov/nuccore/

##       "SalvelinusAlpinus-KJ128605.txt"      ## Arctic Char

## These files are stored in the folder whose full path is assigned to
```

```
## the variable voucherHOME, defined below. The default value assigned
## to this variable is a placeholder that needs to be replaced by the
## appropriate path

######################################################################
## SECTION (BARCODES ASSEMBLER) =======================================
######################################################################

barcodesAssembler =
    function (barcodeFileNames, barcodeLabels, barcodesHOME)
{
    ## DESCRIPTION ============================

    ## To create a labeled list of barcodes corresponding to specified
    ##  specimens. Each barcode is a fragment, about 655 nucleobases
    ##  long, of the cytochrome c oxidase subunit 1 mitochondrial
    ##  gene, MT-CO1

    ## INPUTS =================================

    ##  barcodeFileNames = Character vector with names of plain ASCII
    ##   text files where the character ">" occurs once and once only,
    ##   at the beginning of a line. The lines that follow the sole
    ##   line whose first character is ">" have the barcode proper,
    ##   comprising only letters from {A,C,G,T} and possibly blanc
    ##   spaces

    ## barcodeLabels = Labels to be used as labels of the elements of the
list

    ## barcodesHOME = Full path to the folder containing the files
    ##   named in barcodeFileNames

    ## OUTPUTS ================================

    ## A labeled list whose elements are the barcodes contained in the
    ## files specified in barcodeFileNames, and whose labels are the
    ## character strings in the elements of the vector barcodeLabels

    ## ========================================

    nf = length(barcodeFileNames)
    barcodes = list()
    for (jf in 1:nf)
    {
        r = readLines(con=paste0(barcodesHOME, barcodeFileNames[jf]))
        nr = length(r)
        i = grep("^>", r)
        error = FALSE
        if (length(i) == 0) {
            cat(paste0("## ERROR: File ",
                       barcodeFileNames[jf],
                       " does not have any line ",
                       "that starts with '>'\n"))
```

```
            error = TRUE }
        if (length(i) > 1) {
            cat(paste0("## ERROR: File ",
                       barcodeFileNames[jf],
                       " has multiple lines ",
                       "that start with '>'\n"))
            error = TRUE }
        s = gsub("\\s+", "", paste0(r[(i+1):nr], collapse=""))
        ns = nchar(s)
        a = substring(s, first=1:ns, last=1:ns)
        if (!all(a %in% c("A","C","G","T"))) {
            cat(paste0("## ERROR: Barcode in file ",
                       barcodeFileNames[jf],
                       " has one or more illegal characters\n"))
            error = TRUE }
        barcodes[[jf]] = if (error) {NA} else {s}
    }
    names(barcodes) = barcodeLabels

    return(invisible(barcodes))
}

barcodesHOME="~/NIST/CUSTOMERS/DebraEllisor/PAPER/ABC/SUPPLEMENT/BARCODES
/"

barcodes = barcodesAssembler(
    barcodeFileNames=c(
        "OncorhynchusGorbuscha-FDA245.txt",   ## Pink Salmon
        "OncorhynchusKeta-FDA243.txt",        ## Chum Salmon
        "OncorhynchusKisutch-FDA244.txt",     ## Coho Salmon
        "OncorhynchusMykiss-RFE386.txt",      ## Steelhead Salmon
        "OncorhynchusNerka-FDA242.txt",       ## Sockeye Salmon
        "OncorhynchusTshawytscha-FDA241.txt", ## Chinook Salmon
        "ParahuchoPerryi-JX232184.txt",       ## Sakhalin taimen
        "SalmoSalar-FDA24.txt",               ## Atlantic Salmon
        "SalmoTrutta-JN007797.txt",           ## Brown Trout
        "SalvelinusAlpinus-KJ128605.txt"),    ## Arctic Char
    barcodeLabels=c("Pink", "Chum", "Coho", "Steel", "Sock", "Chin",
                    "Taim", "Atla", "Trut", "Char"),
    barcodesHOME)

######################################################################
## SECTION (SHORT-READ PULLER) =======================================
######################################################################

shortReadPuller =
    function (fastqNAME,
              shortReadSpecification="random",
              shortRead.minLength=0, shortRead.maxLength=Inf,
              fastqHOME)
{
    ## DESCRIPTION ============================

    ## Pulls a short-read and the corresponding sequence of quality
```

```
    ## scores from a specified FASTQ file

    ## INPUTS ===================================

    ## fastqNAME = Name of a FASTQ file

    ## shortReadSpecification = A positive integer I, the string
    ##   "random", or a different character string. If I, then the
    ##   function returns the Ith short-read in the FASTQ file
    ##   (provided I does not exceed the number of short-reads in the
    ##   file). If the string "random", then the function return a
    ##   randomly selected short-read from among those whose length
    ##   lies between shortRead.minLength and shortRead.maxLength. If
    ##   a character string S different from "random", then the
    ##   function returns the short-read whose label is S

    ## shortRead.minLength = Positive integer with the minimum
    ##   length of the short-reads to sample

    ## shortRead.maxLength = Positive integer (or +Inf) with the maximum
    ##   length of the short-reads to sample

    ## fastqHOME = Full path to the folder containing the FASTQ file
    ##   from which to draw short-reads

    ## OUTPUTS ==================================

    ## A short-read pulled from the specified FASTQ file, in the form
    ## of a list whose components are
    ## label = Character string with the label of the short-read
    ## s = Character string with sequence of symbols denoting nucleotides
    ## qc = Character string with the correponding sequence of quality
codes

    ## ========================================

    f = readLines(con=paste0(fastqHOME, fastqNAME))

    if (is.character(shortReadSpecification) &&
        (shortReadSpecification != "random")) {
        js = grep(shortReadSpecification, f)
        if (length(js)==0) {
            stop(paste0("There is no short-read ",
                        "with the specified label\n"), call. = FALSE)
        } else {
            return(list(label=f[js], s=f[js+1], qc=f[js+3]))
        }
    }

    ## Line numbers with first record of each short read: the 1st
    ## record has the label of the short-read; the 2nd record has the
    ## sequence of nucleobases in the short-read; the 3rd record has
    ## the single character "+"; the 4th record has the sequence of
    ## quality scores
```

```
    is = seq(from=1, to=length(f), by=4)
    srLabels = f[is]

    ns = length(is) ## Number of short reads
    nfs = numeric(ns) ## Number of nucleobases in each short read
    for (js in 1:ns) {nfs[js] = nchar(f[is[js]+1])}

    if (tolower(shortReadSpecification) == "random") {
        index = ((shortRead.minLength <= nfs) & (nfs <=
shortRead.maxLength))
        if (sum(index) == 0) {
            stop(paste0("There is no short-read whose length ",
                        "is in specified range\n"), call. = FALSE)
        } else { js = sample((1:ns)[index], size=1) }
    } else {
        if (as.numeric(shortReadSpecification) > ns) {
            stop("Short-read number cannot exceed ", ns, "\n", call. =
FALSE)
        } else {js = shortReadSpecification} }

    return(list(label=f[is[js]], s=f[is[js]+1], qc=f[is[js]+3]))
}

#######################################################################
## SECTION (SHORT-READ SCORER) ========================================
#######################################################################

shortReadScorer =
    function (sr, barcodes, nReplicates=25, nVersions=16, matchesHOME)
{
    ## DESCRIPTION =============================

    ## Given a short-read and corresponding quality scores extracted
    ## from a FASTQ file, and a set of genetic barcodes from reference
    ## specimens, shortReadScorer aligns the short read to each
    ## barcode, does uncertainty evaluation, and outputs a discrete
    ## probability distribution over the set of barcodes that
    ## expresses both the (uncertain) provenance of the short read from a
    ## specimen of the species represented in the set of barcodes, and
    ## the alignment uncertainty

    ## Each barcode is a fragment, about 655 nucleobases long, of the
    ## cytochrome c oxidase subunit 1 mitochondrial gene, MT-CO1.
    ## The alignment is done using MUSCLE (MUltiple Sequence
    ## Comparison by Log-Expectation) version 5.x

    ## The uncertainty evaluation for the alignment to each barcode
    ## recognizes the uncertainty component expressed in MUSCLE's
    ## ensemble bootstrapping, as well as the uncertainty component
    ## resulting from the propagation of the locus-specific quality
    ## scores that express the ambiguity in the identification of
    ## the nucleobases at the different loci

    ## DETAILS ================================
```

```
## The quality scores are translated into locus-specific
## probabilities {pi[l]} of incorrect identification of the
## nucleobases

## K = nReplicates = Number of replicates generated according to
## the error probabilities encoded in the quality scores

## (A) Repeat the following steps for k=1,...,K:

##      (A1) For each locus l, keep the assigned letter with
##      probability pi[l] or replace it with one of the other three
##      letters each with probability (1-pi[l])/3 -- result is matchB

##      (A2) For each barcode jb = 1,...,nb:

##         (A2a) Generate ensemble of nVersions alternative
##               alignments resulting from different perturbations
##               (generated internally within MUSCLE) corresponding
##               to different random number seeds for different
##               versions

##         (A2b) For each version jv = 1,...,nVersions

##               (A2b.1) Assemble sMatch and sRef strings including
##               minus signs (-). These two strings have the same
##               number of characters

##               (A2b.2) Delete all loci of the aligned sequences
##               where there is a minus sign in one or both of
##               sMatch and sRef, obtaining sMatch.clean and
##               sRef.clean as results

##               (A2b.3) Compute the DL distance between
##               sMatch.clean and sRef.clean and store it in
##               D[jb,jv] -- corresponding to barcode jb and
##               version jv (currently this is the raw distance: we
##               may want to replace it with a relative distance)

##      (A3) Jitter the elements of D to break ties randomly and
##      then declare the fish identifications (one per version jv
##      -- that is, per column of D) for the current replicate jr
##      as that fish (that is, barcode, or row of D) that
##      corresponds to the smallest value of in column jv of D

## (B) The relative frequencies of the different fish define the
## probability distribution of the species that the short-read
## originates from

## NOTE: Once such probability distributions will have been
## computed for many short-reads, they can be pooled into a
## consensus distribution that expresses the uncertainty about
## species identification based on all those short-reads
```

```
## INPUTS ==================================

## sr = List with two elements: (i) "s", a string with the
##   sequence of letters from {A,C,G,T} representing the
##   nucleobases in the short-read; (ii) "qc", the corresponding
##   string of quality codes

## barcodes = Named list of barcodes, each of whose elements is a
##   string with the sequence of letters from {A,C,G,T}
##   representing the barcode

## nReplicates = Number of replicated assignments of the
##   short-read to a barcode, each corresponding to a different
##   variant of the short-read according to its error
##   probabilities, with default value 1000

## nVersions = Number of versions of each alignment, produced by
##   MUSCLE's "-diversified" option

## matchesHOME = Folder where temporary files generated during
##   alignment are written

## OUTPUTS =================================

## Table of counts of assignments of the short-read to the species
## represented in the barcodes

## ========================================

require(stringdist)

## Compute the error probabilities for the given short-read, which
## is referred to as "match"

match.nc = nchar(sr$s)
match.atoms = substring(sr$s, first=1:match.nc, last=1:match.nc)
match.QCs = substring(sr$qc, first=1:match.nc, last=1:match.nc)
match.Phreds = numeric(match.nc)
for (jc in 1:match.nc) {
        match.Phreds[jc] = as.numeric(charToRaw(match.QCs[jc]))-33 }
match.ErrorProbs = 10^(-match.Phreds/10)

## Number and labels of the barcodes the short-read will be scored
against

nb = length(barcodes)
barcodeNAMEs = names(barcodes)

fish = array(character(), dim=c(nReplicates, nVersions))
for (jr in 1:nReplicates)
{
    ## Perturb short-read according to error probabilities
    matchB.atoms = match.atoms
    for (jc in 1:match.nc)
```

```
        {
            if (runif(1) < match.ErrorProbs[jc]) {
                alternatives = setdiff(c("A","C","G","T"),
match.atoms[jc])
                matchB.atoms[jc] = sample(alternatives, size=1) }
        }
        ## Bootstrap replicate of short-read
        matchB = paste(matchB.atoms, collapse="")

        ## Generate nVersions of the alignment of the short-read
        ## against each barcode

        ## D[jb,jv] = DL distance from short-read to barcode jb
        ## corresponding to alignment version jv. The smallest value
        ## in each column of D determines the species assigned to the
        ## short-read for alignment version jv
        D = array(dim=c(nb, nVersions))

        for (jb in 1:nb)
        {
            referenceLabel = barcodeNAMEs[jb]
            referenceSequence = barcodes[[jb]]
            matchLabel = ">MATCH"
            matchSequence = matchB

            write(matchLabel, file=paste0(matchesHOME, "match-in.txt"),
                  append=FALSE)
            write(matchSequence, file=paste0(matchesHOME, "match-
in.txt"),
                  append=TRUE)
            write(paste0(">", referenceLabel),
                  file=paste0(matchesHOME, "match-in.txt"),
                  append=TRUE)
            write(referenceSequence, file=paste0(matchesHOME, "match-
in.txt"),
                  append=TRUE)

            ## Generate ensemble of nVersions alternative alignments
            ## for current replicate (jr) that expresses the
            ## uncertainty corresponding to the locus-specific
            ## nucleotide identification error probabilities
            commandString.ensemble =
                paste0("muscle5n -align ", matchesHOME, "match-in.txt",
                       " -quiet -diversified -replicates ", nVersions,
                       " -output ", matchesHOME, "match-out-diversified-
@.txt")
            system(commandString.ensemble)

            versionFileNAMEs =
                list.files(matchesHOME, "^match-out-diversified-
*.*.txt$")
            for (jv in 1:nVersions)
                {
                    ## Mutually aligned short-read and reference
```

```
                    outAlign =
                        readLines(paste0(matchesHOME,
versionFileNAMEs[jv]))

                    ## Separate MATCH and REF at the second occurrence of
">"
                    iSplit = grep(">", outAlign)[2]
                    iMatch.Start = 2
                    iMatch.End = iSplit-1
                    iRef.Start = iSplit+1
                    iRef.End = length(outAlign)

                    ## Assemble MATCH and REF strings including minus
signs (-)
                    ## These strings have the same number of characters
                    sMatch = paste(outAlign[iMatch.Start:iMatch.End],
                                   collapse="")
                    sRef = paste(outAlign[iRef.Start:iRef.End],
                                 collapse="")

                    ## Delete all loci of the aligned sequences where
                    ## there is a minus sign in one or both of sMatch
                    ## and sRef
                    nc = nchar(sMatch)
                    sMatch.atoms = substring(sMatch, first=1:nc,
last=1:nc)
                    sRef.atoms = substring(sRef, first=1:nc, last=1:nc)
                    iKeep = intersect(grep("-", sMatch.atoms,
invert=TRUE),
                                      grep("-", sRef.atoms, invert=TRUE))
                    sMatch.atoms.clean = sMatch.atoms[iKeep]
                    sRef.atoms.clean = sRef.atoms[iKeep]
                    sMatch.clean = paste(sMatch.atoms.clean, collapse="")
                    sRef.clean = paste(sRef.atoms.clean, collapse="")

                    ## NOTE: We may want to change this to relative
distance
                    D[jb,jv] = stringdist(sMatch.clean, sRef.clean,
                                          method="dl")
                }
        }
        ## Jitter D to break ties randomly
        fish[jr,] = barcodeNAMEs[apply(jitter(D), 2, which.min)]
    }
    return(table(fish))
}

######################################################################
## SECTION (SAMPLING & SCORING SHORT-READS) ==========================
######################################################################

system.time(expr={
    nShortReads = 100
    fishCounts = array(rep(0, nShortReads*length(barcodes)),
```

```
                       dim=c(nShortReads, length(barcodes)))
    dimnames(fishCounts)[[2]] = names(barcodes)

    fastqHOME =
"~/NIST/CUSTOMERS/DebraEllisor/PAPER/ABC/SUPPLEMENT/FASTQ/"
    fastqNAME = "CohoSalmonWildCaught-100-200.fastq"
    matchesHOME =
"~/NIST/CUSTOMERS/DebraEllisor/PAPER/ABC/SUPPLEMENT/MATCHES/"
    for (js in 1:nShortReads)
    {
        cat("## ", js, " of ", nShortReads, "\n")
        sr = shortReadPuller(fastqNAME=fastqNAME,
                             shortRead.minLength=100,
                             shortRead.maxLength=200,
                             fastqHOME=fastqHOME)
        fishTable = shortReadScorer(sr, barcodes,
                                    nReplicates=25, nVersions=16,
                                    matchesHOME=matchesHOME)
        fishCounts[js, names(fishTable)] = fishTable
    }
})

######################################################################
## SECTION (POOLING VOTES FOR IDENTIFICATION PROBABILITIES) ==========
######################################################################

z = as.data.frame(fishCounts)

## SIMPLE AVERAGING
cbind(apply(z, 2, sum, na.rm=TRUE) / sum(z, na.rm=TRUE))

## Pink  0.000800  Pink  0.001525
## Chum  0.001225  Chum  0.000325
## Coho  0.924850  Coho  0.944625
## Steel 0.025875  Steel 0.029525
## Sock  0.007500  Sock  0.006925
## Chin  0.013825  Chin  0.008475
## Taim  0.002475  Taim  0.000475
## Atla  0.004800  Atla  0.000200
## Trut  0.016500  Trut  0.007300
## Char  0.002150  Char  0.000625

## ENTROPY-WEIGHTED AVERAGING

z$entropy = apply(z, 1,
                  function (x) {p = x/sum(x, na.rm=TRUE)
                      -sum(p*log(p), na.rm=TRUE)})

z$weights = exp(-2*z$entropy)/sum(exp(-2*z$entropy))

S = 0; for (j in 1:length(barcodes)) {S = S + sum(z[,j]*z$weights)}
probs = cbind(apply(z[,1:length(barcodes)], 2,
                    function (x, w=z$weights) {sum(x*w)})/S)
probs
```

```
## Pink  2.223446e-05  Pink  6.285767e-05
## Chum  6.526643e-05  Chum  7.090248e-06
## Coho  9.780471e-01  Coho  9.820954e-01
## Steel 1.172246e-02  Steel 1.230379e-02
## Sock  3.511056e-04  Sock  6.422384e-04
## Chin  5.717233e-03  Chin  4.076707e-03
## Taim  2.004618e-04  Taim  1.628028e-05
## Atla  1.800021e-04  Atla  4.363230e-06
## Trut  3.542493e-03  Trut  7.569364e-04
## Char  1.516578e-04  Char  3.434672e-05

K = 10000
probsB = array(dim=c(K,length(barcodes)))
dimnames(probsB)[[2]] = dimnames(probs)[[1]]
for (k in 1:K)
{
    iB = sample(1:nrow(z), size=100, replace=TRUE)
    zB = z[iB,]
    zB$entropy =
        apply(zB, 1, function (x) {
            p = x/sum(x, na.rm=TRUE); -sum(p*log(p), na.rm=TRUE)})
    zB$weights = exp(-2*zB$entropy)/sum(exp(-2*zB$entropy))
    SB = 0; for (j in 1:length(barcodes)) {SB = SB +
sum(zB[,j]*zB$weights)}
    probsB[k,] = cbind(apply(zB[,1:length(barcodes)], 2,
                             function (x, w=zB$weights) {sum(x*w)})/SB)
}

results = cbind(MEAN=c(probs), SD=apply(probsB, 2, sd),
                t(apply(probsB, 2, quantile, probs=c(0.025, 0.975))))
dimnames(results)[[1]] = dimnames(probs)[[1]]
results

##               MEAN           SD         2.5%        97.5%
## Pink  2.223446e-05 1.293025e-05 0.000000e+00 5.024936e-05
## Chum  6.526643e-05 4.252540e-05 0.000000e+00 1.635340e-04
## Coho  9.780471e-01 8.804703e-03 9.583982e-01 9.915089e-01
## Steel 1.172246e-02 7.739030e-03 2.341888e-03 2.911071e-02
## Sock  3.511056e-04 1.617746e-04 7.627361e-05 7.097775e-04
## Chin  5.717233e-03 1.619113e-03 2.803420e-03 9.100816e-03
## Taim  2.004618e-04 1.383046e-04 7.774122e-06 5.266781e-04
## Atla  1.800021e-04 1.289382e-04 5.245125e-06 4.779961e-04
## Trut  3.542493e-03 3.271623e-03 5.931150e-05 1.059650e-02
## Char  1.516578e-04 1.010271e-04 7.794146e-06 3.886160e-04

##               MEAN           SD        2.5%        97.5%
## Pink  6.285767e-05 4.439719e-05 0.000000000 1.638134e-04
## Chum  7.090248e-06 7.033115e-06 0.000000000 2.164871e-05
## Coho  9.820954e-01 7.345858e-03 0.965178881 9.931207e-01
## Steel 1.230379e-02 7.063934e-03 0.002436993 2.875121e-02
## Sock  6.422384e-04 5.814529e-04 0.000000000 1.914785e-03
## Chin  4.076707e-03 1.313118e-03 0.001797404 6.900425e-03
## Taim  1.628028e-05 1.152639e-05 0.000000000 4.290894e-05
## Atla  4.363230e-06 4.328071e-06 0.000000000 1.332228e-05
```

```
## Trut  7.569364e-04 7.055080e-04 0.000000000 2.271992e-03
## Char  3.434672e-05 2.624430e-05 0.000000000 9.533054e-05

signif(100*results, 3)
##            MEAN      SD     2.5%    97.5%
## Pink   0.00222 0.00129 0.00e+00  0.00502
## Chum   0.00653 0.00425 0.00e+00  0.01640
## Coho  97.80000 0.88000 9.58e+01 99.20000
## Steel  1.17000 0.77400 2.34e-01  2.91000
## Sock   0.03510 0.01620 7.63e-03  0.07100
## Chin   0.57200 0.16200 2.80e-01  0.91000
## Taim   0.02000 0.01380 7.77e-04  0.05270
## Atla   0.01800 0.01290 5.25e-04  0.04780
## Trut   0.35400 0.32700 5.93e-03  1.06000
## Char   0.01520 0.01010 7.79e-04  0.03890

##            MEAN       SD   2.5%    97.5%
## Pink  6.29e-03 0.004440  0.000  0.01640
## Chum  7.09e-04 0.000703  0.000  0.00216
## Coho  9.82e+01 0.735000 96.500 99.30000
## Steel 1.23e+00 0.706000  0.244  2.88000
## Sock  6.42e-02 0.058100  0.000  0.19100
## Chin  4.08e-01 0.131000  0.180  0.69000
## Taim  1.63e-03 0.001150  0.000  0.00429
## Atla  4.36e-04 0.000433  0.000  0.00133
## Trut  7.57e-02 0.070600  0.000  0.22700
## Char  3.43e-03 0.002620  0.000  0.00953

############################################################################
## ++++++++++++++++++++++++++++++++++++++++++++++++++++++++++++++++++++++ ##
############################################################################
```
